# Causal associations between autoimmune diseases and sarcopenia-related traits: a bi-directional Mendelian randomization study

**DOI:** 10.3389/fgene.2024.1325058

**Published:** 2024-04-04

**Authors:** Chunlan Chen, Ying He

**Affiliations:** ^1^ Department of Pulmonary and Critical Care Medicine, Guangdong Provincial People’s Hospital (Guangdong Academy of Medical Sciences), Southern Medical University, Guangzhou, China; ^2^ Department of Infectious Diseases, The Second Xiangya Hospital of Central South University, Changsha, China; ^3^ Clinical Medical Research Center for Viral Hepatitis in Hunan Province, Changsha, China

**Keywords:** autoimmune diseases, sarcopenia, causal association, Mendelian randomization, genome-wide association studies

## Abstract

**Background:**

Sarcopenia is common in patients with autoimmune diseases (ADs); however, the causal associations between ADs and sarcopenia remain unclear. Therefore, this study investigated the causal associations using bi-directional Mendelian randomization analysis.

**Methods:**

Exposure-related single-nucleotide polymorphisms (SNPs) were extracted from genome-wide association studies (GWASs). GWAS statistics for common ADs [Crohn’s disease (CD), ulcerative colitis (UC), rheumatoid arthritis (RA), systemic lupus erythematosus (SLE), psoriasis (PSO), and multiple sclerosis (MS)] and sarcopenia-related traits [hand grip strength (HGS), appendicular fat-free mass (FFM), and walking pace] were obtained from public datasets. Inverse-variance weighting as the main method was used to evaluate the causal effect.

**Results:**

Genetically predicted CD had causal effects on whole-body FFM (β = −0.005, *p* = 0.001), leg FFM (β_left_ = −0.006, *p* = 1.8E-4; β_right_ = −0.007, *p* = 2.0E-4), and arm FFM (β_left_ = −0.005, *p* = 0.005; β_right_ = −0.005, *p* = 0.001), while RA had causal effects on 8 sarcopenia-related traits, namely, HGS (β_left_ = −2.06, *p* = 2.8E-38; β_right_ = −2.311, *p* = 2E-20), whole-body FFM (β = −0.842, *p* = 4.7E-10), leg FFM (β_left_ = −0.666, *p* = 2.6E-6; β_right_ = −0.073, *p* = 2.1E-3), arm FFM (β_left_ = −0.63, *p* = 4.4E-6; β_right_ = −0.736, *p* = 4.4E-8), and walking pace (β = −1.019, *p* = 6.2E-14). In the reverse direction, HGS (odds ratio [OR]_left_ = 10.257, *p* = 3.6E-5; OR_right_ = 16.445, *p* = 3.7E-7) had causal effects on CD, while HGS (OR_left_ = 0.994, *p* = 0.004; OR_right_ = 0.993, *p* = 1.4E-4), leg FFM (OR_left_ = 1.003, *p* = 0.005; OR_right_ = 1.005, *p* = 1.9E-4), and walking pace (OR = 0.985, *p* = 5.7E-5) were causally associated with RA. No evidence showed causal associations of UC, SLE, PSO, or MS with sarcopenia-related traits.

**Conclusion:**

Our study demonstrated that the genetic susceptibility to CD and RA was associated with high risk of sarcopenia, and some sarcopenia-related traits had causal effects on CD or RA.

## 1 Introduction

Autoimmune diseases (ADs) consist of a wide range of conditions characterized by the breakdown and dysregulation of the immune system, accompanied with various clinical manifestations (Wang et al., 2015). Common ADs include rheumatoid arthritis (RA), Crohn’s disease (CD), ulcerative colitis (UC), systemic lupus erythematosus (SLE), psoriasis (PSO), and multiple sclerosis (MS). Patients with ADs usually suffer from lifelong recurrent symptoms and even finally lose organ function, causing a huge socio–economic burden worldwide (Sparks, 2019; Scherlinger et al., 2020; Griffiths et al., 2021; Olek, 2021; Rogler et al., 2021).

Sarcopenia is characterized by a progressive decrease in muscle mass and function (Cruz-Jentoft and Sayer, 2019). It commonly occurs in geriatrics; however, it also occurs in certain populations, such as patients with chronic inflammatory diseases, cancers, and metabolic disorders (Cruz-Jentoft et al., 2019). Patients with sarcopenia suffer from a poor overall and disease-progression free survival rate, more postoperative complications, longer hospital stays, and higher rates of falls and fractures (Petermann-Rocha et al., 2022).

In recent years, sarcopenia has become increasingly universal in younger patients with ADs, especially RA (An et al., 2020; Bennett et al., 2023). The pathogenesis of sarcopenia in AD patients has not been clarified, but chronic inflammation as the main mechanism in ADs is considered to be a crucial risk factor in sarcopenia (Li et al., 2022). Many studies have reported the prevalence of sarcopenia in AD patients, most of which focused on RA (Bennett et al., 2023). Many researchers conducted cross-sectional studies and demonstrated that the incidence of sarcopenia in RA patients ranged from 10.1% to 45.1%, which was significantly higher than that in controls (Barone et al., 2018; Lin et al., 2019; Mochizuki et al., 2019; Torii et al., 2019; Tournadre et al., 2017). In a study, Santos et al. showed that 16 out of 92 SLE patients (17.4%) had sarcopenia (Santos et al., 2011). Some observational studies showed that the incidence of sarcopenia in UC patients ranged from 14.8% to 69.5% (Adams et al., 2017; Zhang et al., 2017b; Cushing et al., 2018); meanwhile, the incidence of sarcopenia in CD patients ranged from 31.0% to 61.4% (Zhang et al., 2017a; Cravo et al., 2017; Lee et al., 2020). Two observational studies showed that the incidence of sarcopenia in PSO patients ranged from 20% to 40.9% (Krajewska-Wlodarczyk et al., 2017; Barone et al., 2018). In addition, the risk of ADs was also significantly higher in patients with sarcopenia than in controls (An et al., 2020). Collectively, considerable evidence based on observational studies suggested a strong relationship between ADs and sarcopenia; however, observational studies show reverse causality and cannot exclude the effects of confounding factors. Therefore, the causal associations between ADs and sarcopenia remain unclear.

Mendelian randomization (MR) analysis is a genetic epidemiological method used to evaluate the causal relationship between the exposure and outcome, which usually uses independent single-nucleotide polymorphisms (SNPs) extracted from genome-wide association studies (GWASs) as genetic instrumental variables (IVs) (Emdin et al., 2017). Because the genetic makeup is determined when the oosperm is initially formed and is unlikely to be influenced by diseases in later life, the one-way causal effect can be inferred using MR analysis. By eliminating the potential confounders that could influence the outcomes, the MR analysis effectively forms naturally blinded randomized controlled trials (Liu et al., 2022; Ma et al., 2022). Hence, the purpose of this study is to estimate the causal relationships between six common ADs (CD, UC, RA, SLE, PSO, and MS) and sarcopenia-related traits by performing bi-directional two-sample MR analysis.

## 2 Materials and methods

### 2.1 Bi-directional MR study design

**FIGURE 1:**
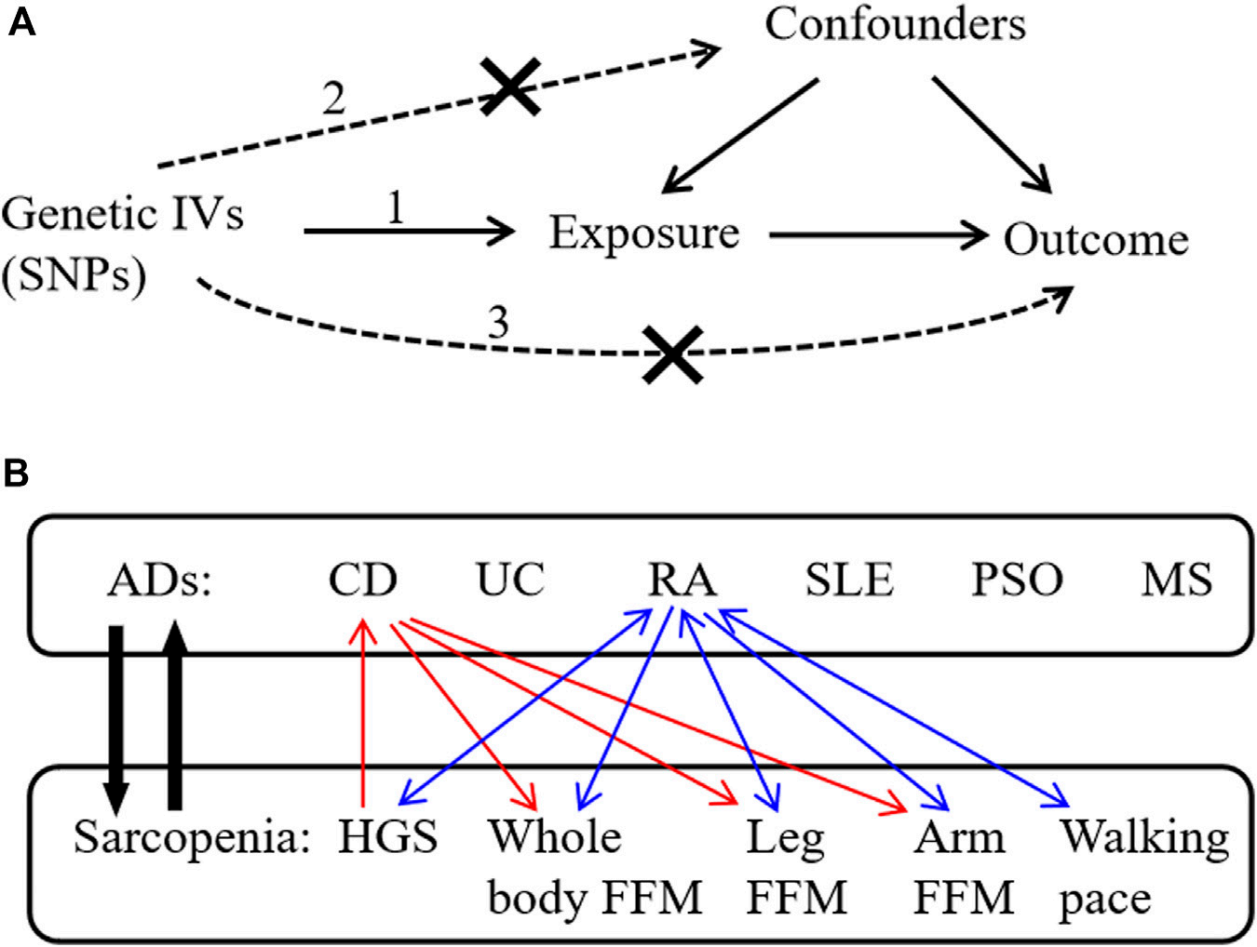
Schematic overview of the study design. **(A)** The three principal assumptions in Mendelian randomization (MR) design are 1) correlation assumption: IVs are strongly related to the exposure; 2) independence assumption: IVs do not affect the outcome through the confounding factors; and 3) exclusion assumption: IVs only affect the outcome via exposure. **(B)** This bi-directional MR analysis was performed to evaluate the causal associations between ADs and sarcopenia-related traits. The red arrows indicate the identified causal associations between CD and sarcopenia-related traits in our results, and the blue arrows indicate the identified causal associations between RA and sarcopenia-related traits in our results. IVs, instrumental variables; SNPs, single-nucleotide polymorphisms; ADs, autoimmune diseases; CD, Crohn’s disease; UC, ulcerative colitis; RA, rheumatoid arthritis; SLE, systemic lupus erythematosus; PSO, psoriasis; MS, multiple sclerosis; HGS, hand grip strength; FFM, fat-free mass.

This study used public GWAS summary data and did not produce or collect any new human data. Since the ethics approval and patient informed consent had already been obtained in the preliminary studies, they were not required for this study. The bi-directional MR analysis was performed to evaluate the causal relationships between six common ADs (CD, UC, RA, SLE, PSO, and MS) and sarcopenia-related traits, including hand grip strength (HGS), whole-body fat-free mass (FFM), leg and arm FFM, and walking pace. The schematic view of this study is shown in Figure 1. In brief, ADs acted as the exposures, while sarcopenia-related traits acted as the outcomes. AD-related SNPs retrieved from the GWAS summary statistics were selected as genetic IVs according to the strict screening criteria. Five MR methods were used to evaluate the causal effects, and subsequently, sensitivity analyses were conducted to verify the reliability and robustness of the results. In addition, reverse MR analysis was conducted to evaluate the causal effects of sarcopenia-related traits as exposures on ADs as outcomes.

### 2.2 Data source

#### 2.2.1 GWAS statistics of six ADs: CD, UC, RA, SLE, PSO, and MS

The GWAS statistics for CD (17,897 cases and 33,977 controls) and UC (13,768 cases and 33,977 controls) of European descent were obtained from the International Inflammatory Bowel Disease Genetics Consortium (IIBDGC) (Liu et al., 2015). The GWAS statistics for RA (5,201 cases and 45,732 controls) and PSO (5,314 cases and 457,619 controls) of European descent were obtained from the UK Biobank (http://www.nealelab.is/uk-biobank/) (Ma et al., 2022). The GWAS statistics for SLE (5,201 cases and 9,066 controls) of European descent were derived from the study by Lv et al. (2022) *.* The GWAS statistics for MS (47,429 cases and 68,374 controls) of European descent were acquired from the International MS Genetics Consortium (IMSGC, 2019).

#### 2.2.2 GWAS statistics of eight sarcopenia-related traits

HGS is widely used as a proxy of muscular fitness to reflect muscle function (Bohannon, 2015). The GWAS statistics for HGS from European individuals, including left HGS (N = 461,026) and right HGS (N = 461,089), were obtained from the UK Biobank, adjusted for hand size (Kuo et al., 2023). Each HGS-related SNP was adjusted for sex, age, age^2^, sex*age, and sex*age^2^ (Sudlow et al., 2015).

FFM is currently considered the most commonly used measure of lean mass, which represents muscle mass and soft tissue mass (Zillikens et al., 2017). The GWAS statistics for sarcopenia-related FFM of European individuals comprise five traits: whole-body FFM (N = 454,850), left leg FFM (N = 454,805), right leg FFM (N = 454,835), left arm FFM (N = 454,672), and right arm FFM (N = 454,753) (Larsson et al., 2020). These five GWAS statistics from the UK Biobank were measured using the bioelectrical impedance analysis method and adjusted for sex, age, age^2^, sex*age, and sex*age^2^ (Sudlow et al., 2015).

Gait speed is another important diagnostic indicator for sarcopenia. Sarcopenia can be diagnosed by low muscle mass, low muscle strength, and/or low gait speed (≤0.8 m/s), based on the diagnostic consensus proposed by the European Working Group on Sarcopenia in Older People (EWGSOP) (Cruz-Jentoft et al., 2010). Walking pace can be used as an indicator of low gait speed in sarcopenia research. The GWAS statistics of walking pace (N = 459,915 European individuals) were obtained from the UK Biobank (Ma et al., 2022).

All GWAS statistics for ADs and sarcopenia-related traits could be freely downloaded on the IEU OpenGWAS database project (https://gwas.mrcieu.ac.uk/). The details of these datasets are given in Supplementary Table S1.

### 2.3 Genetic IV selection

To obtain qualified SNPs as genetic IVs, a series of strict screening steps was used. Three assumptions must be met in MR analysis (Emdin et al., 2017): (1) correlation assumption: genetic IVs are strongly related to the exposure; (2) independence assumption: IVs cannot influence the outcome through the confounding factors; and (3) exclusion assumption: IVs only affect the outcome via exposure (Figure 1).

For the correlation assumption, the following standards were required: (1) significant association (F > 10, *p* < 5 × 10^−8^) of genome-wide SNPs with the exposure. The F-statistic of a SNP was calculated by the equation (Thompson and Burgess, 2015) F = [β/se]^2^, where β represents the effect size and se represents the standard error of β; (2) linkage disequilibrium (LD) clumping (*r* ^2^ < 0.001, window size = 1 Mb) was used to screen independent SNPs. For the independence and exclusion assumption, each significant exposure-related SNP was checked using PhenoScanner (http://www.phenoscanner.medschl.cam.ac.uk/), and then, SNPs associated with the potential confounders were eliminated. The confounders that may affect sarcopenia and ADs include smoking, body mass index, physical inactivity, malnutrition, and extreme sleep duration (Baurecht et al., 2021; Freuer et al., 2022). The harmonization procedure was subsequently used to ensure the SNPs with a minor allele frequency (>0.01), unify the effect direction and effect allele, and eliminate the palindromic and incompatible SNPs. Finally, the MR-Pleiotropy RESidual Sum and Outlier (MR-PRESSO) test was adopted to detect the horizontal pleiotropy and outliers of SNPs in the MR analysis (Verbanck et al., 2018).

### 2.4 MR analysis

Five MR methods were used in our study: inverse-variance weighted (IVW), weighted median, MR–Egger, maximum likelihood, and penalized weighted median (Thompson and Burgess, 2015). The IVW method was used as the key method as it has the best efficiency for causal estimation (Borges et al., 2022). The estimation by the IVW method is consistent and close to the true effect, when the pleiotropy is not significant and the sample size of IVs is sufficient (Bowden et al., 2016). If no significant heterogeneity (IVW-derived Cochran’s Q statistic *p*-value ≥0.05) was observed, the fixed effect of the IVW method was used; otherwise, the random effect was used. Because unbalanced pleiotropy may lead to potential bias in the causal estimation using the IVW method, supplementary MR methods and sensitivity analyses are required to confirm the robustness of causal estimation (Burgess et al., 2019). MR–Egger, weighted median, maximum likelihood, and penalized weighted median were used as supplementary MR methods to confirm the causal estimation obtained by the IVW method (Thompson and Burgess, 2015; Burgess and Thompson, 2017). Despite of lower statistical power [wider confidence interval (CI)], the supplementary methods can provide stronger and more credible causal estimates over a wider range of scenarios.

To adjust for multiple tests and avoid false-positive results, the Bonferroni correction was adopted using a corrected *p*-value (0.05/n, where n represents the number of exposure factors) to reach the significance level (Li et al., 2023). For MR estimates from six ADs to sarcopenia-related traits, *p*-value <0.008 (0.05/6) was set as significant, and *p*-value <0.006 (0.05/8) was considered significant in MR estimates from eight sarcopenia-related traits to ADs.

The R package (version 4.2.1), two-Sample MR package (version 0.5.6), and MR-PRESSO package (version 1) were used in the statistical analyses.

### 2.5 Sensitivity analysis

The potential horizontal pleiotropy of SNPs was assessed using the MR–Egger method. MR–Egger intercept *p*-value ≥0.05 indicates no significant pleiotropy. The heterogeneity was assessed using Cochran’s Q statistic in the IVW method. Cochran’s Q statistic *p*-value ≥0.05 indicates no significant heterogeneity.

## 3 Results

### 3.1 Causal effects of ADs on sarcopenia-related traits

IVW and four supplementary MR methods were used for estimating causal effects of 6 ADs on 8 sarcopenia-related traits; hence, we tested a total of 48 causality pairs. According to the established quality control criteria (F > 10, *p* < 5 × 10^−8^, *r* ^2^ < 0.001), AD-related SNPs were selected as confounder-independent IVs (Supplementary Tables S2–S7). After LD clumping and harmonization, the MR-PRESSO test was conducted to detect SNP outliers. After the removal of SNP outliers, we finally obtained eligible IVs (94–100 SNPs for CD, 64–70 SNPs for UC, 5–6 SNPs for RA, 28–34 SNPs for SLE, 15–19 SNPs for PSO, and 48–54 SNPs for MS) for the following MR analysis with sarcopenia-related traits (Supplementary Table S8).

Since sarcopenia-related traits as outcomes are continuous variables, we used the β-value as the estimate effect. As mentioned previously, we used a Bonferroni-corrected *p*-value of 0.008 (0.05/6) as significance. If the heterogeneity test showed Cochran’s Q statistic *p*-value <0.05, the random effect of IVW was applied; otherwise, the fixed effect of IVW was applied. The IVW results demonstrated that genetically predicted CD is associated with a higher risk of whole-body FFM [β = −0.005, 95% CI = (−0.007, −0.003), *p* = 0.001], leg FFM [β_left_ = −0.006, 95% CI = (−0.010, −0.002), *p* = 1.8E-4; β_right_ = −0.007, 95% CI = (−0.010, −0.002), *p* = 2.0E-4], and arm FFM [β_left_ = −0.005, 95% CI = (−0.009, −0.001), *p* = 0.005; β_right_ = −0.005, 95% CI = (−0.009, −0.001), *p* = 0.001], while genetically predicted CD has no causal effect on HGS [β_left_ = −0.003, 95% CI = (−0.007, 0.001), *p* = 0.096; β_right_ = −0.002, 95% CI = (−0.006, 0.002), *p* = 0.203] and walking pace [β = −0.001, 95% CI = (−0.005, 0.003), *p* = 0.616] (Table 1).

**TABLE 1:** Association estimates for CD on sarcopenia-related traits.

Exposure	Outcome	No. of IVs	Heterogeneity	Pleiotropy	MR result		
			Cochran’s Q (*p*)	MR–Egger	Method	β (95% CI)	*p*
				intercept *p*			
CD	Left HGS	94	181.85 (<0.001)	0.615	IVW	−0.003 (−0.007, 0.001)	0.096
					Weighted median	−0.0003 (−0.004, 0.004)	0.886
					MR–Egger	−0.001 (−0.011, 0.009)	0.878
CD	Right HGS	95	169.52 (<0.001)	0.979	IVW	−0.002 (−0.006, 0.002)	0.203
					Weighted median	−0.0005 (−0.004, 0.003)	0.825
					MR–Egger	−0.002 (−0.010, 0.006)	0.651
CD	Whole-body FFM	94	215.69 (<0.001)	0.88	IVW	−0.005 (−0.007, −0.003)	0.001*
					Weighted median	−0.004 (−0.008, −0.001)	0.015
					MR–Egger	−0.006 (−0.014, 0.002)	0.17
CD	Left leg FFM	94	198.68 (<0.001)	0.384	IVW	−0.006 (−0.010, −0.002)	1.8E-4*
					Weighted median	−0.007 (−0.011, −0.003)	**1.9E-4**
					MR–Egger	−0.01 (−0.018, −0.002)	0.027
CD	Right leg FFM	94	204.54 (<0.001)	0.385	IVW	−0.006 (−0.010, −0.002)	2.0E-4*
					Weighted median	−0.007 (−0.011, −0.003)	**4.5E-4**
					MR–Egger	−0.01 (−0.018, −0.002)	0.027
CD	Left arm FFM	95	207.51 (<0.001)	0.599	IVW	−0.005 (−0.009, −0.001)	0.005*
					Weighted median	−0.006 (−0.010, −0.002)	0.002*
					MR–Egger	−0.007 (−0.015, 0.001)	0.122
CD	Right arm FFM	96	226.84 (<0.001)	0.656	IVW	−0.005 (−0.009, −0.001)	0.001*
					Weighted median	−0.007 (−0.011, −0.003)	**2.5E-4**
					MR–Egger	−0.007 (−0.015, 0.001)	0.104
CD	Walking pace	100	240.83 (<0.001)	0.092	IVW	−0.001 (−0.005, 0.003)	0.616
					Weighted median	0.001 (−0.003, 0.005)	0.732
					MR–Egger	0.006 (−0.002, 0.014)	0.17

Bonferroni-corrected significance level of *p*-value <0.008 (0.05/6) was used to adjust for multiple tests. The bold *p*-value labeled with asterisk denotes the significant association. The β-value denotes the estimate from the MR analysis. CD, Crohn’s disease; HGS, hand grip strength; FFM, fat-free mass; MR, Mendelian randomization; IVs, instrumental variables; CI, confidence interval; IVW, inverse-variance weighted. The bold *p*-value labeled with asterisk meant the significant association.

Notably, genetically predicted RA is associated with 8 sarcopenia-related traits, HGS [β_left_ = −2.06, 95% CI = (−2.372, −1.748), *p* = 2.8E-38; β_right_ = −2.311, 95% CI = (−2.795, −1.827), *p* = 2E-20], whole-body FFM [β = −0.842, 95% CI = (−1.107, −0.577), *p* = 4.7E-10], leg FFM [β_left_ = −0.666, 95% CI = (−0.944, −0.388), *p* = 2.6E-6; β_right_ = −0.073, 95% CI = (−1.195, −0.265), *p* = 2.1E-3], arm FFM [β_left_ = −0.63, 95% CI = (−0.899, −0.361), *p* = 4.4E-6; β_right_ = −0.736, 95% CI = (−0.999, −0.473), *p* = 4.4E-8], and walking pace [β = −1.019, 95% CI = (−1.284, −0.754), *p* = 6.2E-14] (Table 2). However, the IVW results suggested that genetic susceptibility to UC, SLE, PSO, or MS is not associated with the risk of the eight sarcopenia-related traits (Supplementary Table S8). No significant horizontal pleiotropy was detected using MR–Egger analysis, which supported the robustness and reliability of the IVW-derived estimates. Overall, our MR analyses demonstrated that genetic susceptibility to CD has significant causal effects on FFM of the whole body, legs, and arms, while RA has significant causal effects on all eight sarcopenia-related traits.

**TABLE 2:** Association estimates for RA on sarcopenia-related traits.

Exposure	Outcome	No. of IVs	Heterogeneity	Pleiotropy	MR result		
			Cochran’s Q (*p*)	MR–Egger intercept (*p*)	Method	β (95% CI)	*p*

RA	Left HGS	6	10.33 (0.066)	0.948	IVW	−2.06 (−2.372, −1.748)	2.8E-38*
					Weighted median	−2.106 (−2.500, −1.712)	1.3E-25*
					MR–Egger	−2.086 (−2.974, −1.198)	0.001*
RA	Right HGS	6	12.07 (0.033)	0.336	IVW	−2.311 (−2.795, −1.827)	**1.0E-20**
					Weighted median	−2.421 (−2.807, −2.035)	1.4E-34*
					MR–Egger	−2.7 (−3.545, −1.855)	0.003*
RA	Whole-body FFM	5	3.577 (0.466)	0.669	IVW	−0.842 (−1.107, −0.577)	4.7E-10*
					Weighted median	−0.829 (−1.125, −0.533)	3.6E-8*
					MR–Egger	−0.734 (−1.263, −0.205)	0.073
RA	Left leg FFM	5	7.7 (0.103)	0.48	IVW	−0.666 (−0.944, −0.388)	2.6E-6*
					Weighted median	−0.666 (−0.997, −0.335)	8.6E-05*
					MR–Egger	−0.399 (−1.163, 1.561)	0.382
RA	Right leg FFM	5	11.19 (0.024)	0.624	IVW	−0.73 (−1.195, −0.265)	2.1E-3*
					Weighted median	−0.788 (−1.100, −0.476)	7.2E-7*
					MR–Egger	−0.5 (−1.470, 0.470)	0.386
RA	Left arm FFM	5	1.83 (0.765)	0.555	IVW	−0.63 (−0.899, −0.361)	4.4E-6*
					Weighted median	−0.58 (−0.892, −0.268)	2.7E-4*
					MR–Egger	−0.483 (−0.993, 0.027)	0.16
RA	Right arm FFM	5	1.89 (0.755)	0.586	IVW	−0.736 (−0.999, −0.473)	4.4E-8*
					Weighted median	−0.718 (−1.028, −0.408)	5.7E-6*
					MR–Egger	−0.495 (−0.995, −0.005)	0.098
RA	Walking pace	6	3.03 (0.695)	0.39	IVW	−1.019 (−1.284, −0.754)	6.2E-14*
					Weighted median	−1.046 (−1.377, −0.715)	7.2E-10*
					MR–Egger	−0.827 (−1.299, −0.355)	0.026

A Bonferroni-corrected significance level of *p*-value <0.008 (0.05/6) was used to adjust for multiple tests. The bold *p*-value labeled with an asterisk denotes the significant association. The β-value denotes the estimate from the MR analysis. RA, rheumatoid arthritis; HGS, hand grip strength; FFM, fat-free mass; MR Mendelian randomization; IVs, instrumental variables; CI, confidence interval; IVW, inverse-variance weighted. The bold *p*-value labeled with asterisk meant the significant association.

### 3.2 Causal effects of sarcopenia-related traits on ADs

For estimating the causal effects of 8 sarcopenia-related traits on 6 ADs, our study also tested a total of 48 causality pairs. According to the established quality control criteria, SNPs of eight sarcopenia-related traits were selected as confounder-independent IVs (Supplementary Tables S9–S16). After LD clumping, harmonization, and removal of SNP outliers, we finally obtained eligible IVs (5–144 SNPs for left HGS, 7–157 SNPs for right HGS, 21–496 SNPs for whole-body FFM, 22–452 SNPs for left leg FFM, 24–458 SNPs for right leg FFM, 24–496 SNPs for left arm FFM, 25–463 SNPs for right arm FFM, and 3–54 SNPs for walking pace) for the following MR analysis with six ADs (Supplementary Tables S17).

Since ADs as outcomes are classified variables, we used the odds ratio (OR) as the estimate effect. As mentioned previously, a Bonferroni-corrected *p*-value of 0.006 (0.05/8) was considered significant. The IVW results demonstrated that genetic susceptibility to HGS is associated with the risk of CD [OR_left_ = 10.257, 95% CI = (3.396, 30.983), *p* = 3.6E-5; OR_right_ = 16.445, 95% CI = (5.585, 48.422), *p* = 3.7E-7]; however, the other six sarcopenia-related traits have no causal effect on CD (Table 3). Notably, the IVW results showed that HGS [OR_left_ = 0.994, 95% CI = (0.990, 0.998), *p* = 0.004; OR_right_ = 0.993, 95% CI = (0.990, 0.997), *p* = 1.4E-4], leg FFM [OR_left_ = 1.003, 95% CI = (1.001, 1.005), *p* = 0.005; OR_right_ = 1.005, 95% CI = (1.003, 1.007), *p* = 1.9E-4], and walking pace [OR = 0.985, 95% CI = (0.977, 0.993), *p* = 5.7E-5] are causally associated with RA, while whole-body FFM and arm FFM have no causal effect on RA (Table 4). In addition, the IVW results suggested that genetic susceptibility to eight sarcopenia-related traits is not associated with the risk of UC, SLE, PSO, or MS (Supplementary Tables S17). Significant horizontal pleiotropy was not observed in the MR–Egger analysis, which supported the robustness and credibility of the IVW-derived estimates. Taken together, our MR analysis demonstrated that HGS has significant causal effects on CD; meanwhile, HGS, leg FFM, and walking pace have significant causal effects on RA.

**TABLE 3:** Association estimates for sarcopenia-related traits on CD.

Exposure	Outcome	No. of IVs	Heterogeneity	Pleiotropy	MR result		
			Cochran’s Q (*p*)	MR–Egger intercept (*p*)	Method	OR (95% CI)	*p*

Left HGS	CD	5	5.63 (0.227)	0.9	IVW	10.257 (3.396, 30.983)	3.6E-5*
					Weighted median	6.190 (1.854, 20.920)	0.003*
					MR–Egger	3.504 (0.497, 24.733)	0.452
Right HGS	CD	4	3.84 (0.279)	0.431	IVW	16.445 (5.585, 48.422)	3.7E-7*
					Weighted median	12.305 (3.277, 46.829)	2.0E-4*
					MR–Egger	2.413 (0.302, 19.309)	0.071
Whole-body FFM	CD	15	50.48 (<0.001)	0.221	IVW	0.782 (0.301, 2.031)	0.613
					Weighted median	1.251 (0.545, 2.897)	0.597
					MR–Egger	2.784 (0.324, 23.953)	0.368
Left leg FFM	CD	16	31.35 (0.007)	0.618	IVW	1.514 (0.693, 3.310)	0.298
					Weighted median	1.956 (0.844, 4.574)	0.117
					MR–Egger	2.455 (0.325, 18.555)	0.399
Right leg FFM	CD	15	33.74 (0.002)	0.779	IVW	1.730 (0.716, 4.179)	0.222
					Weighted median	1.941 (0.827, 4.592)	0.127
					MR–Egger	2.307 (0.264, 20.161)	0.463
Left arm FFM	CD	17	40.112 (<0.001)	0.73	IVW	1.679 (0.783, 3.598)	0.183
					Weighted median	2.312 (1.041, 5.175)	0.04
					MR–Egger	2.264 (0.360, 14.232)	0.397
Right arm FFM	CD	18	45.82 (0.0002)	0.893	IVW	0.874 (0.414, 1.844)	0.722
					Weighted median	0.757 (0.366, 1.579)	0.454
					MR–Egger	0.796 (0.169, 3.745)	0.776
Walking pace	CD	3	20.02 (<0.001)	0.443	IVW	3.939 (0.794, 19.537)	0.113
					Weighted median	0.072 (0.006, 0.919)	0.041
					MR–Egger	0.643 (0.106, 3.886)	0.422

A Bonferroni-corrected significance level of *p*-value <0.006 (0.05/8) was used to adjust for multiple tests. The bold *p*-value labeled with an asterisk denotes the significant association. CD, Crohn’s diseases; HGS, hand grip strength; FFM, fat-free mass; MR, Mendelian randomization; IVs, instrumental variables; CI, confidence interval; OR, odds ratio; IVW, inverse-variance weighted. The bold *p*-value labeled with asterisk meant the significant association.

**TABLE 4:** Association estimates for sarcopenia-related traits on RA.

Exposure	Outcome	No. of IVs	Heterogeneity	Pleiotropy	MR result		
			Cochran’s Q (*p*)	MR–Egger intercept *p*	Method	OR (95% CI)	*p*
				intercept *p*			
Left HGS	RA	140	189.44 (0.003)	0.583	IVW	0.994 (0.990, 0.998)	0.004*
					Weighted median	0.995 (0.989, 1.001)	0.051
					MR–Egger	0.999 (0.983, 1.015)	0.873
Right HGS	RA	153	173.22 (0.114)	0.061	IVW	0.993 (0.990, 0.997)	1.4E-4*
					Weighted median	0.995 (0.989, 1.001)	0.003*
					MR–Egger	1.006 (0.992, 1.020)	0.393
Whole-body FFM	RA	488	626.02 (<0.001)	0.308	IVW	1.003 (1.001, 1.004)	0.008
					Weighted median	1.004 (1.002, 1.006)	0.008
					MR–Egger	1.005 (1.001, 1.009)	0.048
Left leg FFM	RA	451	601.87 (<0.001)	0.113	IVW	1.003 (1.001, 1.005)	0.005*
					Weighted median	1.005 (1.003, 1.007)	1.9E-4*
					MR–Egger	1.006 (1.002, 1.010)	0.011
Right leg FFM	RA	457	588.94 (<0.001)	0.089	IVW	1.003 (1.001, 1.005)	0.002*
					Weighted median	1.005 (1.001, 1.009)	2E-4*
					MR–Egger	1.007 (1.001, 1.013)	0.006*
Left arm FFM	RA	465	603.19 (<0.001)	0.223	IVW	1.003 (1.001, 1.005)	0.014
					Weighted median	1.003 (1.001, 1.005)	0.079
					MR–Egger	1.005 (0.999, 1.011)	0.04
Right arm FFM	RA	462	591.18 (<0.001)	0.497	IVW	1.003 (1.001, 1.005)	0.007
					Weighted median	1.003 (0.999, 1.007)	0.071
					MR–Egger	1.004 (0.998, 1.010)	0.102
Walking pace	RA	52	48.61 (0.568)	0.926	IVW	0.985 (0.977, 0.993)	5.7E-5*
					Weighted median	0.983 (0.974, 0.993)	0.001*
					MR–Egger	0.987 (0.955, 1.021)	0.447

A Bonferroni-corrected significance level of *p*-value <0.006 (0.05/8) was used to adjust for multiple tests. The bold *p*-value labeled with an asterisk denotes the significant association. RA, rheumatoid arthritis; HGS, hand grip strength; FFM, fat-free mass; MR, Mendelian randomization; IVs, instrumental variables; CI, confidence interval; OR, odds ratio; IVW, inverse-variance weighted. The bold *p*-value labeled with asterisk meant the significant association.

## 4 Discussion

In this study, we used GWAS summary statistics to investigate the causal associations between ADs and sarcopenia-related traits by conducting a bi-directional two-sample MR analysis. Our study identified the significant causal effects of CD on FFM of the whole body, legs, and arms and, in the reverse direction, the significant causal effect of HGS on CD. Moreover, our results revealed that RA and sarcopenia-related traits had significant causal effects on each other. Collectively, our study suggested that the genetic susceptibility to CD or RA is associated with the risk of sarcopenia, and also, the genetic susceptibility to sarcopenia is associated with the risk of CD or RA. However, no evidence for causal associations between the other ADs (UC, SLE, PSO, and MS) and sarcopenia-related traits was observed. To the best of our knowledge, this is the first bi-directional MR study to explore the causal associations between ADs and sarcopenia-related traits.

Malnutrition, a major contributor to sarcopenia, is very prevalent in patients with CD or UC. CD patients are more likely to have severe malnutrition and sarcopenia than UC patients (Scaldaferri et al., 2017). A recent meta-analysis showed that the prevalence of sarcopenia in patients with CD or UC is 52% or 37% (Ryan et al., 2019), respectively. This may be because the impaired main site of nutrient absorption, extensive mucosal lesions, fistulas, or gastrointestinal obstruction is more common in patients with CD (Nishikawa et al., 2021). Our MR analysis showed that CD, but not UC, has significant causal effects on FFM of the whole body, legs, and arms, which supported the clinical observation of a higher prevalence of sarcopenia in patients with CD. FFM is currently considered the most widely used index of muscle mass, and the original definition of sarcopenia was only focused on the loss of muscle mass. Hence, FFM, especially that of leg and arm, can reflect the status of sarcopenia well. Although our results showed that CD has no causal effect on HGS and walking pace, the significant causal effect of CD on leg and arm FFM meant that CD is positively associated with the loss of muscle mass rather than the loss of muscle function. However, a comprehensive assessment of sarcopenia, including muscle function, is necessary for CD patients as our results showed that HGS has significant causal effects on CD, which suggested that reduced muscle function may be positively associated with the poor prognosis of CD. The mechanisms driving sarcopenia in CD patients include malabsorption, vitamin D deficiency, chronic inflammation in the gut, adipose tissue, and muscle–gut axis (Dhaliwal et al., 2021; Nardone et al., 2021; Nishikawa et al., 2021). Therefore, interventions targeting the above factors should be helpful in reducing the incidence of sarcopenia in CD patients. In addition, due to the significant side effects caused by immunosuppressor or biologic agents, precision treatment based on pharmacogenomics is more and more important for patients with CD. Identification of genes that are sensitive or resistant to biologic agents will provide optimal options for future precision treatment of CD patients with sarcopenia. Increasing studies show that patients with CD carrying the HLA-DQA1*05 allele are at a high risk of low infliximab concentrations or developing immunogenicity to infliximab (Solitano et al., 2023). Furthermore, tumor necrosis factor-α (TNF-α) gene polymorphisms are associated with disease susceptibility and response to etanercept (TNF-α inhibitor) in psoriatic arthritis patients (Murdaca et al., 2014; Murdaca et al., 2017). In short, clinicians should pay more attention to the relationship between CD and sarcopenia and promote the early and comprehensive screening of sarcopenia in CD patients.

RA is a systemic and chronic autoimmune-mediated inflammatory disease characterized by destructive inflammation in multiple synovial joints (Sparks, 2019). Numerous studies have shown that sarcopenia is a significant comorbidity in RA patients (Tournadre et al., 2017; Barone et al., 2018; Lin et al., 2019; Mochizuki et al., 2019; Torii et al., 2019). A recent meta-analysis, consisting of 17 studies, indicated that the pooled prevalence of sarcopenia in RA patients was 31% (Li et al., 2021). Another meta-analysis (including 16 studies from the United States, Europe, Asia, North Africa, Turkey, and New Zealand) showed that the pooled prevalence of sarcopenia in RA patients was 30.2% (Dao et al., 2021). Previous studies suggested that there might be a bi-directional relationship between muscle mass and inflammatory arthritis since the changes in muscle mass such as elevated amino acid catabolism and unbalance between oxidative metabolism and glycolysis are closely related to greater disease activity of RA (Andonian et al., 2021). However, this idea has not been confirmed due to the lack of high-quality prospective cohort studies. Based on the bi-directional MR analysis, our study demonstrated that a mutual causal relationship exists between RA and some sarcopenia-related traits (HGS, leg FFM, and walking pace). Although the definition of sarcopenia differs in different groups, loss of muscle function (HGS and walking pace) and muscle mass (leg and arm FFM) has been widely considered the significant traits of sarcopenia (Larsson et al., 2019). Our study strongly suggested that genetic susceptibility to RA is significantly associated with the risk of low muscle function and muscle mass, which supports that RA patients are susceptible to sarcopenia. In addition, our MR study also suggested that genetic susceptibility to muscle function and muscle mass (leg FFM) is causally associated with RA. The risk factors for driving sarcopenia in RA patients include old age, visceral fat, physical inactivity, malnutrition, extreme sleep duration, body mass index, low protein intake, glucocorticoid usage, and joint damage (Bennett et al., 2023). The pro-inflammatory cytokines (such as interleukin-6 and TNF-α) produced during RA development are associated with proteolysis and resting energy expenditure, which are contributors to sarcopenia (Bennett et al., 2023). Overall, our MR analysis provided evidence that a mutual causal interaction exists between RA and sarcopenia, but mechanisms mediating the interaction still need more investigations.

Our study has three main strengths. First, this study used more comprehensive and new GWAS data to estimate the causal associations between ADs and sarcopenia-related traits using rigorous MR analysis with the exclusion of potential confounders. Second, our study adopted six common ADs and eight sarcopenia-related traits, including muscle mass and muscle function, which could provide more reliable results with potential clinical implications. Third, the GWAS statistics for both ADs and sarcopenia-related traits were obtained from European individuals, which would avoid the bias caused by different ethnic populations. However, some potential limitations should not be neglected. First, limitations of the key assumptions in MR analysis should be noted since it is difficult to guarantee the exclusion of all potential confounders. Second, ADs have diverse severity, age, and drug use; however, stratification analyses are not viable due to using summary statistics, which may lead to biased results. Third, individuals with ADs or sarcopenia-related traits came from different medical units, and the heterogeneity in diagnosis may generate bias. Fourth, since all the GWAS data were obtained from Europeans, the generalizability of the results to other ethnic groups was limited. Finally, because the MR analysis was used to evaluate the causality from the genetic level, we could analyze the potential causal association between the exposure and outcome but not clarify the specific biological mechanisms causing this causality. Biological mechanisms by which ADs affect the sarcopenia-related traits remain to be explored by more basic and clinical research studies.

## 5 Conclusion

This study demonstrated that genetic susceptibility to CD and RA was causally associated with a high risk of sarcopenia, and some sarcopenia-related traits also had causal effects on CD or RA. The causal relationships between CD/RA and sarcopenia may provide a genetic explanation as to why patients with CD or RA are prone to developing sarcopenia. In addition, our results emphasized the importance of early screening of sarcopenia in patients with CD or RA, which may be helpful for the intervention of these diseases.

## Data Availability

The original contributions presented in the study are included in the article/Supplementary Material; further inquiries can be directed to the corresponding author.
